# Wild *Termitomyces* Species Collected from Ondo and Ekiti States Are More Related to African Species as Revealed by ITS Region of rDNA

**DOI:** 10.1100/2012/689296

**Published:** 2012-05-02

**Authors:** Victor Olusegun Oyetayo

**Affiliations:** Department of Microbiology, Federal University of Technology, P.M.B. 704, Akure, Nigeria

## Abstract

Molecular identification of eighteen *Termitomyces* species collected from two states, Ondo and Ekiti in Nigeria was carried out using the internal transcribed spacer (ITS) region. The amplicons obtained from rDNA of *Termitomyces* species were compared with existing sequences in the NCBI GenBank. The results of the ITS sequence analysis discriminated between all the *Termitomyces* species (obtained from Ondo and Ekiti States) and *Termitomyces* sp. sequences obtained from NCBI GenBank. The degree of similarity of T1 to T18 to gene of *Termitomyces* sp. obtained from NCBI ranges between 82 and 99 percent. *Termitomyces* species from Garbon with ascension number AF321374 was the closest relative of T1 to T18 except T12 that has T. eurhizus and T. striatus as the closet relative. Phylogenetic tree generated with ITS sequences obtained from NCBI GenBank data revealed that T1 to T18 are more related to *Termitomyces* species indigenous to African countries such as Senegal, Congo, and Gabon.

## 1. Introduction

*Termitomyces* species belongs to a group called “termitophilic Agaricales” This group was created for these fungi by Heim [1]. There is symbiosis association that exists between the termite and the fungus, *Termitomyces*, since neither of the two partners can exist without the other. Hence, artificial cultivation had been difficult. *Termitomyces species *is a well known edible mushroom in Nigeria.

These mushrooms make their appearance after heavy rains [2] and grow in contact with termite nests in forest soil. They usually appear between the months of April through October. *Termitomyces* species is an important source of enzymes of industrial importance such as xylanase, amylase, and cellulase [3], antioxidant compounds such as polyphenol and vitamin C [4]; protein (31.4–36.4%) [5] and immunostimulatory agent [6]. There is evidence that the extract can activate splenocytes [6].

For a long time, most researchers in Nigeria examine mushrooms with the naked eye based on phenotypic characters. It has been impossible to distinguish between genetically related species by this method. Morphologically, mushrooms belonging to different genera may look similar. The present study mainly focuses on ascertaining the phylogenetic relationship between *Termitomyces* species found in Ondo and Ekiti States Nigeria by sequencing of their ITS zone. Moreover, comparing the gene sequence of *Termitomyces* species from Ondo and Ekiti States Nigeria with sequences obtained from the NCBI GeneBank.

## 2. Materials and Methods

### 2.1. Fungal Material

**Figure 1:**
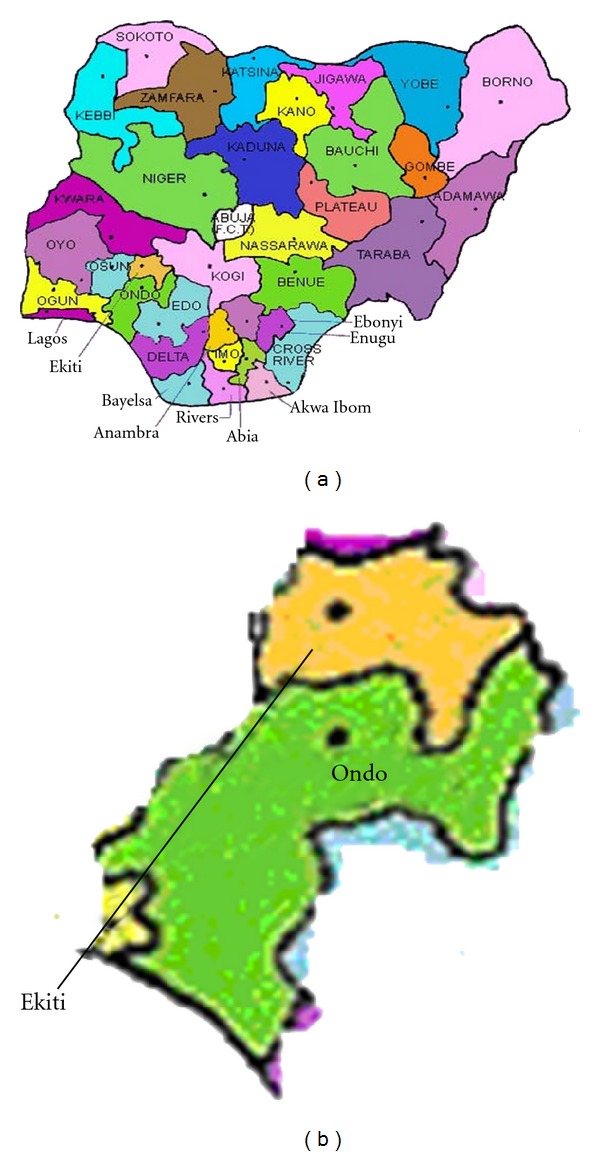
Map of Nigeria (a) and map of the states Ondo and Ekiti States (b) where *Termitomyces* samples were collected.

Fungal material Fresh fruiting body of Termitomyces species were collected from Ekiti and Ondo States (Figure 1), Nigeria (Table 1). The fruitbodies were kept dry by wrapping in tissue paper and keeping in a polythene paper containing silica gel. The polythene bags containing the samples were well labeled for easy identification.

### 2.2. Extraction of DNA

Standard DNA isolation methods employing CTAB lysis buffer [7] was used. Briefly, dried *Termitomyces* fruitbodies were ground in mortal. The grinded materials were transferred into well-labeled tube. Prewarmed extraction buffer (CTAB) was added, and the tubes were incubated at 65°C for 30 to 60 minutes. Equal volume of chloroform and alcohol (24 : 1) was added and mixed by inverting tubes for 15 minutes. The tubes were centrifuged for 10 minutes at 10,000 g (13000 rpm). The process was repeated, but the time of mixing was 3 minutes and time of centrifugation was 5 minutes at the same speed as above. Upper aqueous layers were removed into clean tubes, and 40 *μ*L NaAc was added followed by 260 *μ*L of cold isopropanol. This was gently mixed by inverting tubes. The tubes were incubated at −20°C overnight. On the second day, the mixture was centrifuged at 10,000 g (13000 rpm) for 10 minutes. The supernatant was discarded and pellets rinsed with 70% alcohol and mixed for sometimes. This procedure was repeated three times. After discarding the supernatant, the sample was dried in a dryer for 20 minutes at room temperature. Pellets were resuspended in 30 *μ*L TE. DNA concentration and quality was checked on an ethidium-stained agarose gel (0.7%) using 0.2 *μ*L of each sample.

### 2.3. PCR Amplification of the ITS Region

The entire region of ITS4 and ITS5 was amplified by PCR. The reaction mix was made up to a total volume of 25 *μ*L, composed of 23 *μ*L of *Taq *polymerase “Ready to Go” (Pharmacia) with 0.2 *μ*L of each primer (100 pM) and 2 *μ*L of DNA solution. The tubes were placed in a thermal cycler (GenAmp PCR System 2400; Perkin-Elmer) for amplification under the following conditions: 30 cycles of (1) denaturation at 95°C for 30 s, (2) annealing at 50°C for 1 min, (3) extension at 72°C for 1 min. The amplification products were purified using a PCR Purification Kit and electrophoresed on agarose gel. The amplified products were purified using a PCR Purification Kit and electrophoresed on ethidium-stained agarose gel (0.7%) to check the purity. DNA sequencing was performed using the primers (ITS 4 and ITS 5) in an Applied Biosystem DNA Analyser.

### 2.4. Sequencing of DNA and Alignment of Sequence

Alignments were performed with the Clustal W package [8]. The aligned sequences were corrected manually, focusing on gap positions. DNA sequence data were analyzed to provide pairwise percentage sequence divergence. The data obtained from the sequence alignment were used to plot a tree diagram (Tree View, Win 32).

## 3. Results and Discussion

The results of the ITS sequence analysis discriminated between all the 18 *Termitomyces* species obtained from Ondo and Ekiti States, Nigeria (T1 to T18) and *Termitomyces* sp. sequences obtained from NCBI GenBank. The ITS region of the rDNA is the most used genomic region for molecular characterization of fungi [9, 10] (Gardes and Bruns, 1993). The degree of similarity of T1 to T18 to gene of *Termitomyces* sp. Obtained from NCBI ranges between 82 and 99 percent (Table 2). *Termitomyces* species from Garbon with ascension number AF321374 was the closest relative of T1 to T18 (Table 3).

**Figure 2:**
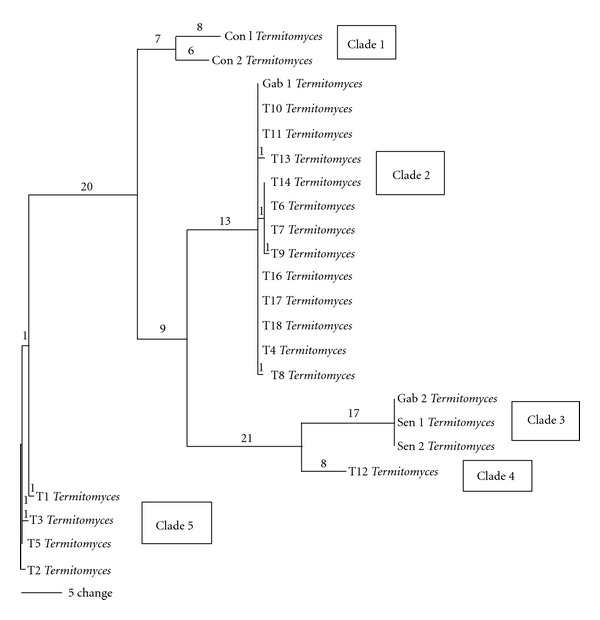
Phylogenetic tree showing positions of *Termitomyces *species collected from Akure and Ado Ekiti (T1 to T18) relative to existing sequences obtained from NCBI Genbank ITS sequence data.

Phylogenetic tree generated with ITS sequences obtained from NCBI GenBank data base revealed that T1 to T18 are more related to *Termitomyces* species indigenous to African countries such as Senegal, Congo, and Gabon (Figure 2). Five clades were observed in the final phylogenetic tree; Clade 1 was made up of *Termitomyces* species (Con 1 and Con 2) from Congo DR. Clade 2 was made up of *Termitomyces* species (Gab 1) from Gabon and *Termitomyces* species (T4, T7, T8, T9, T10, T11, T12, T13, T14, T16, T17, and T18) from Nigeria. This implies that the *Termitomyces* species from Nigeria and Gab 1 may be from the same ancestral stock. Clade 3 was made up of *Termitomyces* species from Gabon (Gab2) and Senegal (Sen 1 and 2). Clade 4 was made up of only *Termitomyces* species T12 while clade 5 was made up of *Termitomyces* species (T1, T2, T3, and T5) from Nigeria. This suggests that they may be new species. 

 The closest relatives of T1 to T18 which were phenotypically identified as *T. clypeatus* and *T. robustus* were *T. striatus* and *T. eurhizus* except T15 which was *T. microcarpus* as revealed by BLAST search (Table 2). Earlier report by Frøslev et al. [11] showed that *Sinotermitomyces carnosus*, *S. griseus *and *S. rugosiceps* are synonyms of *T. mammiformis*. Moreover, Frøslev et al. [11] also found that *S. cavus *and *S. taiwanensis* are, respectively, conspecific with *T. heimii *and *T. clypeatus*. Another study by Oyetayo [12] revealed that phylogenetic tree generated from the ITS sequence obtained from *Termitomyces* species earlier identified phenotypically as *T. clypeatus *was found to be 100% homologous to *T. eurhizus *found in NCBI GenBank. This shows that *T. clypeatus* from Nigeria may be conspecific of *T. eurhizus*.

This study showed that not all the gene sequence of *Termitomyces* species indigenous to Nigeria are 100% homologous with existing gene sequences in NCBI GenBank. *Termitomyces* species from some countries in Africa such as Congo, Gabon, and Senegal are more closely related to *Termitomyces* species indigenous to Nigeria. This may suggest common origin. An earlier phylogenetic study of some African *Termitomyces *revealed that they are from monophyletic origin [13]. Clades 4 and 5 shows that *Termitomyces* species (T1, T2, T3, T5, and T12) are totally different from others species whose gene sequences are already in NCBI GenBank.

## Figures and Tables

**Table 1:** Information on *Termitomyces* species collected from Ondo and Ekiti States.

*Termitomyces* sp.	Location where collection was made	State	Year of collection	Name of collector
T1	Ado Ekiti	Ekiti	October, 2006	Oyetayo, V. O.
T2	Ado Ekiti	Ekiti	October, 2006	Oyetayo, V. O.
T3	FUT, Akure	Ondo	September, 2006	Oyetayo, V. O.
T4	FUT, Akure	Ondo	September, 2006	Oyetayo, V. O.
T5	Ado Ekiti	Ekti	September, 2006	Oyetayo, V. O.
T6	Akure	Ondo	September, 2006	Oyetayo, V. O.
T7	Aule	Ondo	October, 2007	Fakoya, S.
T8	Aule	Ondo	October, 2007	Fakoya, S.
T9	Igbatoro	Ondo	July, 2009	Oyetayo, V. O.
T10	Igbatoro	Ondo	July, 2009	Oyetayo, V. O.
T11	UNAD Road	Ekiti	October, 2009	Oyetayo, V. O.
T12	Orita Obele, Akure	Ondo	July, 2009	Oyetayo, V. O.
T13	Obanla, FUTA	Ondo	September, 2008	Oyetayo, V. O.
T14	Orita Obele, Akure	Ondo	September, 2009	Fakoya, S.
T15	Ilara Mokin	Ondo	August, 2009	Fakoya, S.
T16	Ogbese	Ondo	August, 2009	Fakoya, S.
T17	Owena	Ondo	August, 2009	Fakoya, S.
T18	Obanla, FUTA	Ondo	September, 2009	Fakoya, S.

**Table 2:** Genomic identification based on the ITS gene sequences of *Termitomyces* species collected from Ondo and Ekiti States, Nigeria.

Termitomyces	Phenotypic identity	Closest relative in NCBI GenBank	Ascension number of closest relative	% Identity with sequence from NCBI GenBank
T1	*T. clypeatus*	*T. striatus*	AB073519	89
T2	*T. clypeatus*	*T. striatus*	AF321367	91
T3	*T. robustus *	*T. eurhizus*	AF321366	91
T4	*T. robustus*	*T. striatus*	AF321367	93
T5	*T. rubustus*	*T. striatus*	AB073519	89
T6	*T. robustus*	*T. striatus*	AF321374	93
T7	*T. clypeatus*	*T. striatus*	AF321367	93
T8	*T. robustus *	*T. striatus*	AB073519	93
T9	*T. clypeatus*	*T. striatus*	AF321367	91
T10	*T. clypeatus*	*T. striatus*	AF321367	93
T11	*T. clypeatus*	*T. striatus*	AF321367	93
T12	*T. clypeatus*	*T. eurhizus*	AB073529	88
T13	*T. clypeatus*	*T. striatus*	AF321374	98
T14	*Termitomyces sp.*	*T. striatus*	AF321374	99
T15*	*T. microcarpus*	*T. microcarpus*	AB073529	82
T16	*Termitomyces sp.*	*T. striatus*	AF321374	99
T17	*Termitomyces sp.*	*T. striatus*	AF321374	99
T18	*Termitomyces sp.*	*T. striatus*	AF321374	99

*Phenotypic identification confirmed with genomic data.

**Table 3:** Information on gene sequence of *Termitomyces* species from NCBI GenBank with close identity with T_1_ to T_18_.

Ascension number	Name	Location
AB073519	*Termitomyces* sp. group3	Thailand: Saraburi
AF321367	*Termitomyces striatus *	Republic of Congo
AF321366	*Termitomyces eurhizus *	Republic of Congo
AF321374**	*Termitomyces *sp. AGI	Gabon
AB073529	*Termitomyces* sp. group 8	Thailand: Khao Kitchagoot
AF321364	*Termitomyces* sp. OSI	Senegal
AF321365	*Termitomyces* sp. ASI	Senegal

**Gene sequence of *Termitomyces* sp. from NCBI GenBank with the closest identity with most *Termitomyces* sp. from Ondo and Ekiti states, Nigeria.
